# LIS–lnterlink—connecting laboratory information systems to remote primary health–care centres via the Internet

**DOI:** 10.1155/S1463924698000091

**Published:** 1998

**Authors:** Barry Clark, Bartosz Wachowiak, Ewan W. Crawford, Zenon Jakubowski, Janusz Kabata

**Affiliations:** 1 Glasgow University Department of Pathological Biochemistry Gartnavel General Hospital 1053 Gt Western Rd, Glasgow G12 0YN UK; 2 Nova Medical Polska Al. Rzeczpospolitej, 33 Gdansk 80-463 Poland

## Abstract

A pilot study was performed to evaluate the feasibility of using the Internet to securely deliver patient laboratory results, and the system has subsequently gone into routine use in Poland. The system went from design to pilot and then to live implementation within a four-month period, resulting in the LIS-Interlink software product. Test results are retrieved at regular intervals from the BioLink^TM^ LIS (Laboratory Information System), encrypted and transferred to a secure area on the Web server. The primary health-care centres dial into the Internet using a local-cell service provided by Polish Telecom (TP), obtain a TCP/IP address using the TP DHCP server, and perform HTTP ‘get’ and ‘post’ operations to obtain the files by secure handshaking. The data are then automatically inserted into a local SQL database (with optional printing of incoming reports)for cumulative reporting and searching functions. The local database is fully multi-user and can be accessed from different clinics within the centres by a variety of networking protocols.


Journal of Automatic Chemistry, Vol. 20, No. 3 (May-June 1998) pp. 77-81

### LIS-Interlink—connecting laboratory information systems to remote primary health-care centres via the Internet

**Barry Clark**
*Glasgow University, Department of Pathological Biochemistry, Gartnavel General Hospital, 1053 Gt Western Rd, Glasgow G12 0YN, UK*

**Bartosz Wachowiak**
*Nova Medical Polska, Al. Rzeczpospolitej, 33, 80-463, Gdansk, Poland*

**Ewan W. Crawford**
*Glasgow University, Department of Pathological Biochemistry, Gartnavel General Hospital, 1053 Gt Western Rd, Glasgow G12 0YN, UK*

**Zenon Jakubowski and Janusz Kabata**
*Nova Medical Polska, Al. Rzeczpospolitej, 33, 80-463, Gdansk, Poland*

*A pilot study was performed to evaluate the feasibility of using the Internet to securely deliver patient laboratory results, and the system has subsequently gone into routine use in Poland. The system went from design to pilot and then to live implementation within a four-month period, resulting in the LIS-Interlink software product. Test results are retrieved at regular intervals from the BioLink™ LIS (Laboratory Information System), encrypted and transferred to a secure area on the Web server. The primary health-care centres dial into the Internet using a local-cell service provided by Polish Telecom (TP), obtain a TCP/IP address using the TP DHCP server, and perform HTTP 'get' and 'post' operations to obtain the files by secure handshaking. The data are then automatically inserted into a local SQL database (with optional printing of incoming reports) for cumulative reporting and searching functions. The local database is fully multi-user and can be accessed from different clinics within the centres by a variety of networking protocols.*

### Materials and methods

### *Laboratory Information System*

The Laboratory Information System (LIS) is the multi-disciplinary, multi-lingual BioLink™ LIS from Glasgow University (contact Dr Clark for more details). BioLink™ is a client-server LIS currently running on Btrieve/Scaleable SQL (Pervasive Software, 8834 Capital of Texas Highway N, Suite 300, Austin, Texas 8759, USA; http://www.pervasive.com), supporting Netware IPX/SPX, TCP/IP and NETBUI protocols. BioLink™ is highly fault-tolerant, making it ideal for situations where there can be problems with network cabling and power supplies. The current release version of BioLink™ uses Turbo Pascal (Borland International Inc, 100 Borland Way, Scotts Valley, CA 95066, USA; http://www.borland.com) with embedded SQL.

### *LIS-Interlink software*

The LIS-Interlink software (LS) was developed using RAD (Rapid Application Design) tools and specific Web components, with embedded SQL (Scaleable SQL, Pervasive Software).

*Rapid Application Development (RAD) Environment*: The RAD development environment consisted of Delphi-1 and Delphi-2 (Borland) and Wavetools-32 version 2.0 (Transcom Software Inc, e-mail: sales@transerve.com, http://www.transerve.com). Wavetools are a set of native Delphi components, and the principal component used was TWeb, which creates and handles HTTP protocol connections over TCP/IP enabled networks. The component approach minimizes installation and support problems as there are no OCXs, external DLLs or other external software components, and results in high performance. Delphi-1 was used because Scaleable SQL (Pervasive Software) only supported local hard-disk databases under a 16-bit environment, whereas the Wavetools components required a 32-bit environment (Delphi-2). Because of this temporary complexity both BioLink™ and LIS-Interlink are in the process of being linked to the Interbase (Borland) universal database engine, running on top of Windows™ NT SQL-Server™ (Microsoft Corporation, One Microsoft Way, Redmond, WA 98052-6399, USA), but this change is only cosmetic, so that the RAD can be moved to Delphi-3.

*Software operation*: The system for transmitting results is divided into two sections as shown in figure 1. The BioLink™ side periodically updates the Web server file until the file is collected by the remote host. To prevent conflicts during the actual file transfer, a semaphore system is used as shown in figure 2. This approach is simple and effective and is based on the semaphores for multi-tasking environments, and forms the basis of the secure links which have been previously made between BioLink™ and the CareSys (UniSys, Unisys Ltd, City Gate, 22-24 Southwark Bridge Road, London SE1 9HB, UK; http://www.unisys.com) and MDIS (McDonnell Information Systems Limited, Boundary Way, Hemel Hempstead, Hertfordshire HP2 7HU, UK) Hospital Information Systems for ward-based requesting. When new test results are available they are fetched from the appropriate BioLink™ databases and, after encryption, stored in files on the Web server. Each result line contains hospital and ward information and this information is used to append the encrypted test result to the appropriate file. After an authorized (i.e. password protected)

0142-0453/98 $12·00 © 1998 Taylor & Francis Ltd

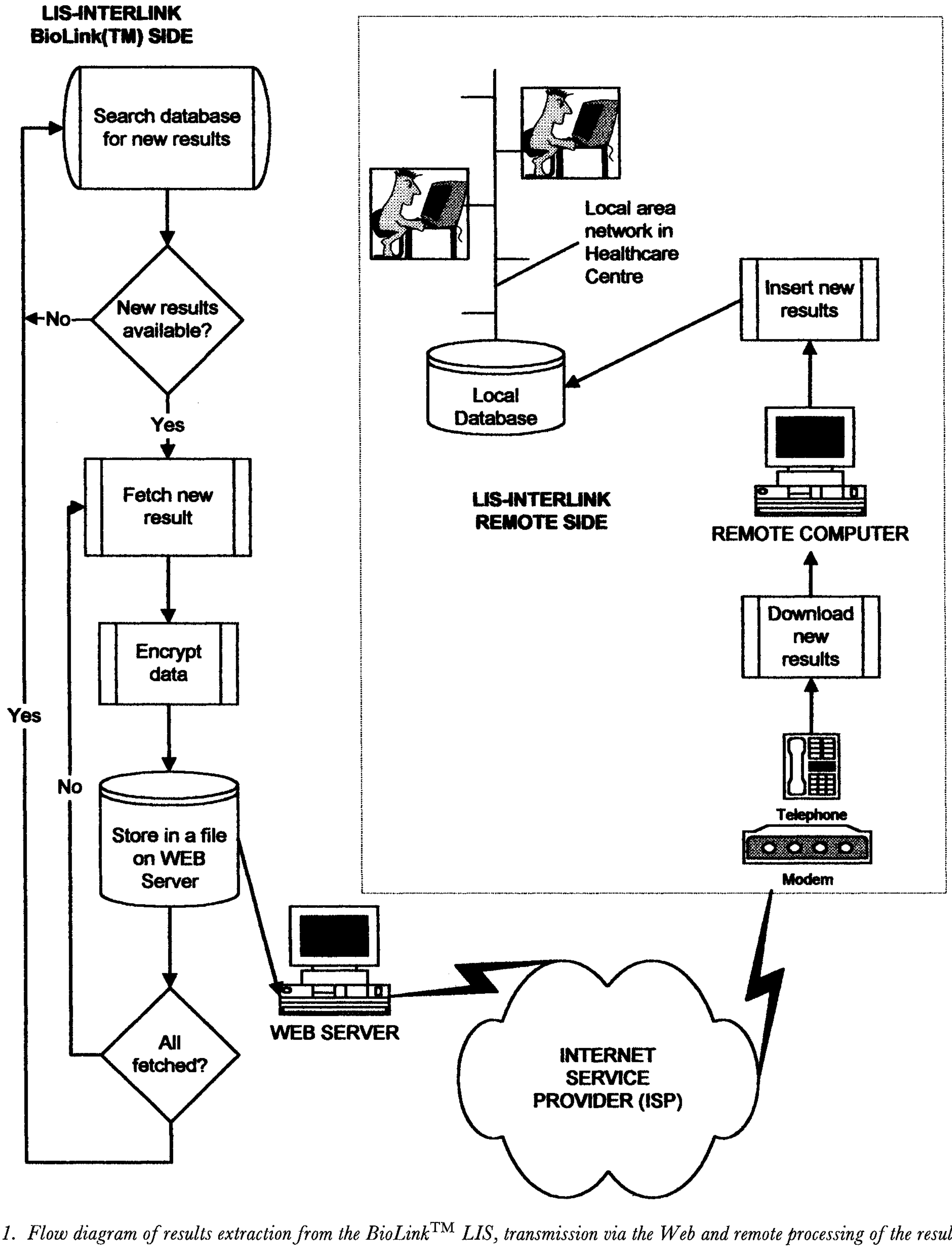

*Figure 1. Flow diagram of results extraction from the BioLink™ LIS, transmission via the Web and remote processing of the results files.*

download has occurred, the file is deleted to avoid duplication. A function on the BioLink™ side of LIS-Interlink allows users to download complete sets of results between a range of dates, in the event of a remote database failure (or damage to the remote computer), so that missing data can be rebuilt.

At the remote side, the user attaches periodically to the ISP, simply loading the remote LIS-Interlink client by double-clicking a short-cut icon on the desktop. The Wavetools TWeb component knows if it is connected to a live TCP/IP node or not, and, if not, automatically instructs Windows™ '95 to use its dial-up facility to make the link. Once connected, the user enters a password, and the file containing the test results is downloaded from the laboratory Web server. After downloading the file is automatically decrypted and all of the results are inserted into the local database. Each

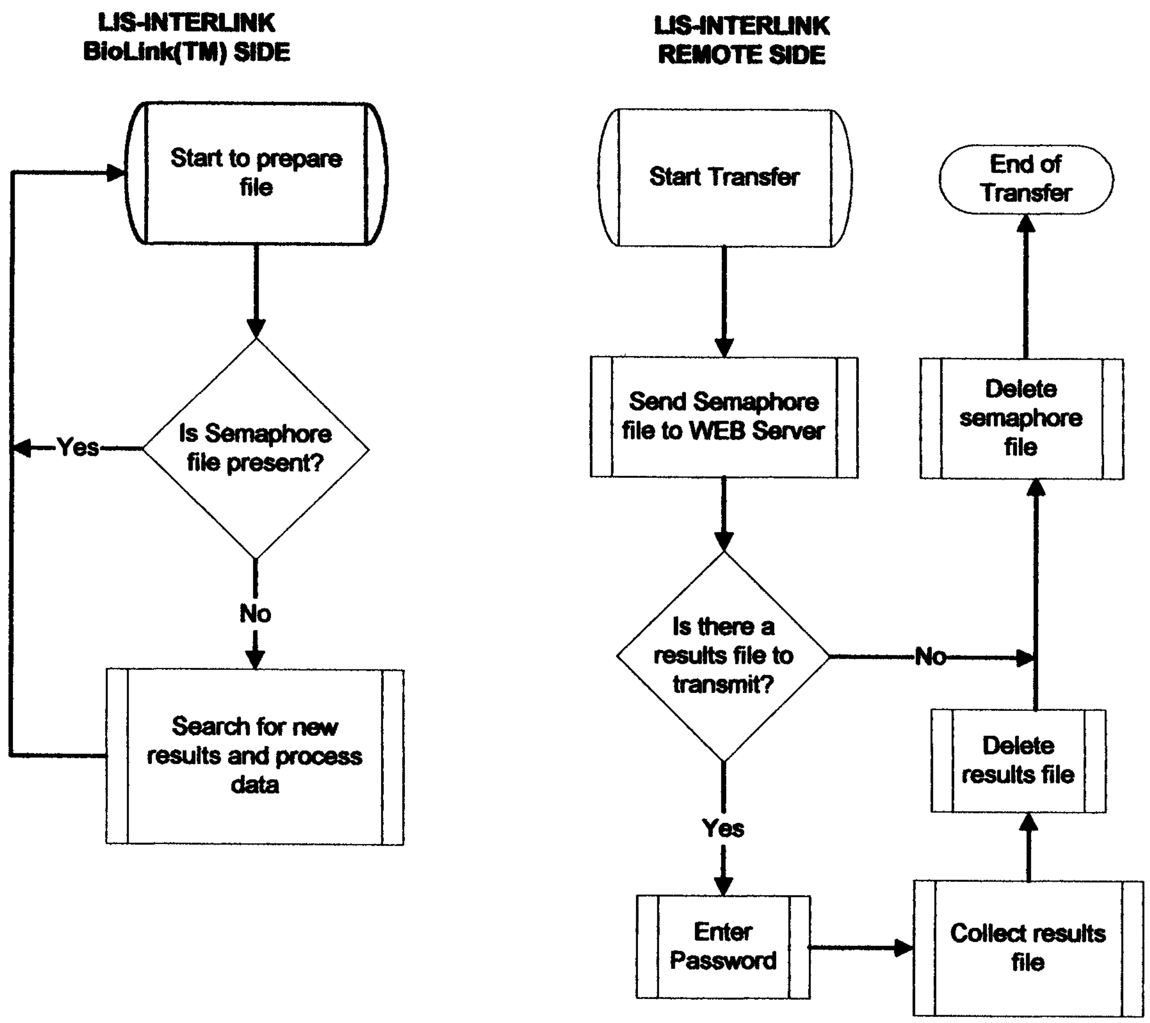

*Figure 2. Flow diagram of the process of semaphore interlocking to ensure file transfer integrity.*

patient has a unique identifier, which ensures that cumulative results are available to the doctors, improving patient care. A typical patient search screen on the remote database is shown in figure 3. In the event of multiple samples for a patient, these are displayed in the sample date list, and the doctor simply clicks on the date required in order to display the results. Clicking on 'profiles' enables the doctor to see if any work is outstanding (BioLink$^{TM}$ ships a list of requested profiles with each results transmission), and clicking on 'comments' allows display of any diagnostic information entered by the laboratory medical staff. Hard copies of the reports can be printed on demand. All screen prompts are stored in an .INI file facilitating translation into any Windows$^{TM}$ supported language. The software uses the basic fonts which are installed by Windows$^{TM}$ so that LIS-Interlink is able to automatically use the appropriate language font without need for programming changes.

There are three levels of protection against possible errors in the download file structure. First, the software generates a checksum (CRC–cyclic redundancy check using an XOR of each byte in the string) for each result line string. Second, because every result consists of definite fields that are of definite type and might have other additional features (like specific length or limited range of values), the integrity of a result is also checked based on these features. Third, if there are errors which have not been detected by the CRC, it is then unlikely that the decryption algorithm would work correctly, as the resultant key generated from the line in error would be incorrect, causing subsequent decryptions to fail; this state is alerted to the user, the process cancelled, so that the remote user could repeat the download.

*Encryption/decryption*: The data encryption/decryption algorithms are those set out in ANSI X9.19 1986 (Financial Institution Retail Message Authentication) which must be used in conjunction with the Data Encryption Algorithm (DEA) as defined in ANSI X3.92-1981—reaffirmed 1987 (American National Standards Institute, 11 West 42nd Street New York, NY 10036, USA). The DEA cryptographic key consists of 64 bits: 56 random and independent bits must be used as the active key, and the other eight bits are used as parity bits to detect errors within the key (bits 8, 16, 24, ..., 64). The parity of every 8-bit byte should be odd (i.e. the number of 1-bits including the parity bit should be odd). Once a line of data has been encrypted, the DEA returns a residual 64-bit MAC key. This is processed by a One Way Function (OWF) in conjunction with the 64-bit MAC key in order to generate a new MAC key for the next results line. The

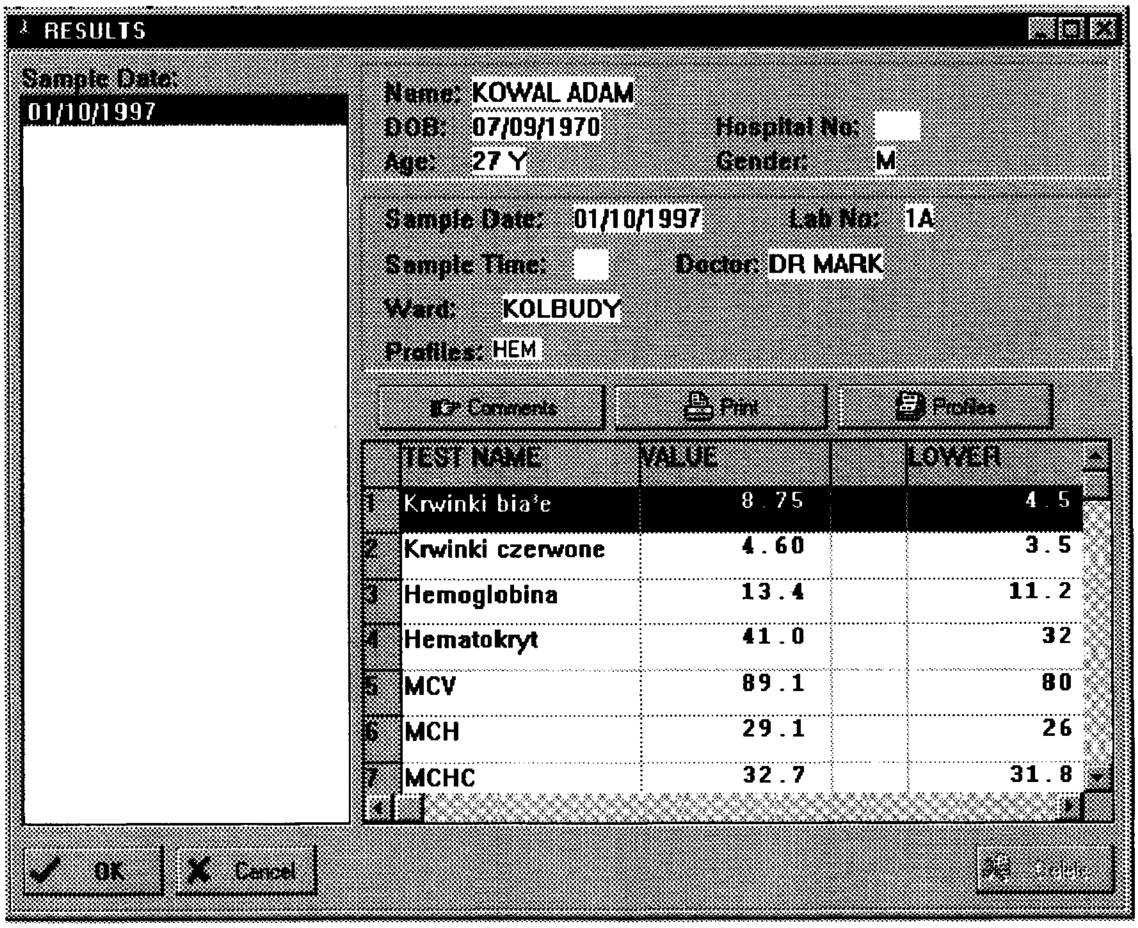

*Figure 3. Screen shot of the LIS-Interlink doctor's patient search screen.*

OWF process is defined in the APACS Standards Manual in Part-3, Page 60 (Association for Payment Clearing Services, Mercury House, Triton Court, 14 Finsbury Square, London EC2 1BR).

The encrypted file consists of following segments:

(1) Name of the ward + check byte.
(2) Additional bytes which index the first encryption key.
(3) Result 1 + CRC.
(4) Result 2 + CRC.
(5) ...
(6) result $n$ + CRC.

The file is encrypted using the 64-bit key. Every file is encoded using different set of starter (or seed) keys. Figure 4 shows the procedure for processing each test result. Decryption on the remote side is precisely opposite to the encryption process.

*Web server*

The Web server was OmniHTTPd version 1.01 (Omnicron Technologies Corporation, 1338—11th Avenue S.W., Calgary, Alberta, Canada T3C 0M6; http://www.fas.harvard.edu/~glau/omnicron/contact.html) installed on an Intel 100 MHz Pentium-based PC with 32 Mb RAM, 2.0 Gb hard-disk and a 3 com network card, running under Windows™ '95. The Web server was connected to the BioLink™ file-server as a standard Novell Netware connection.

*Remote dial-up connection*

The Web server was connected to a US-Robotics courier modem [3Com (UK) Ltd Capitol House, Waterfront Quay, Salford Quays, Manchester M5 2XW; http://www.3com.com] via the serial port. Once 'modems' has been configured on the Windows™ 'settings' screen, dial-up networking needs to be installed from the Windows™ '95 CD/disks and then the 'My computer' icon is used to configure the adapter. A TCP/IP protocol needs to be attached to the adapter and set to get a TCP/IP address automatically. Other information required by the TCP/IP protocol has to be obtained from the ISP. The TWeb component used in LIS-Interlink knows how to interact with the dial-up network adapter automatically, and the dial-up adapter also knows how to correctly handle error conditions, e.g. line busy, no answer, no modem carrier, etc.

### Case study

LIS-Interlink is currently implemented in Nova Medical Polska, Gdansk, Poland. Several local health centres (20–

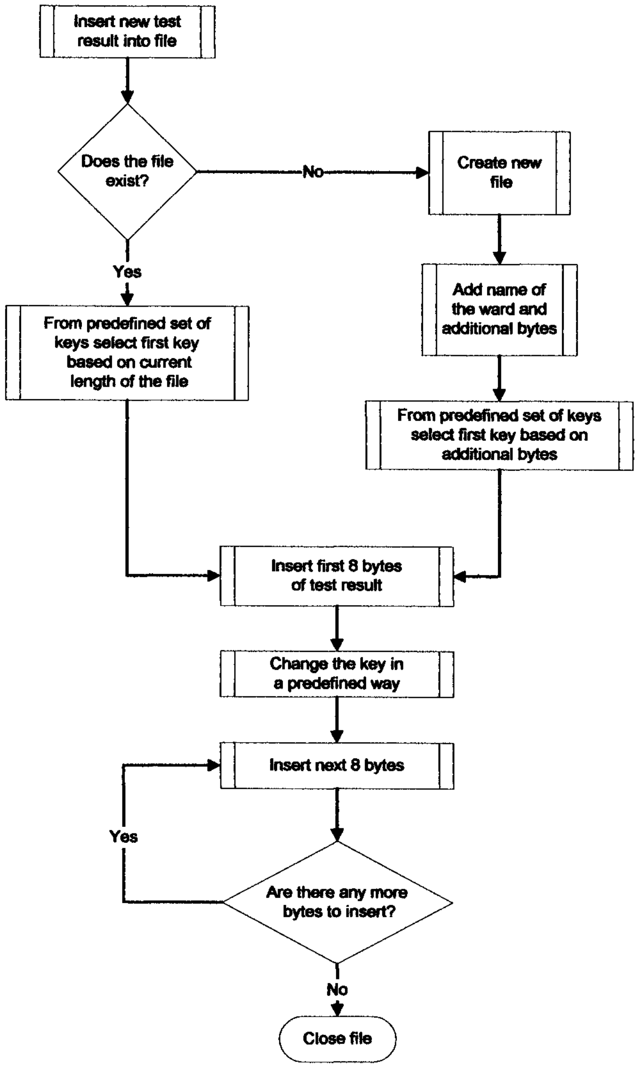

*Figure 4. Flow diagram of the results encryption process.*

50 km away from the laboratory) are already on line (Sukova and Kolbudy), and they use it for getting patient results from Nova Medical laboratory in Gdansk. The file size for 30–40 patients is about 40 kb and downloading such files takes in the order of 40–60 s at 14.4 K. Inserting that number of patient result records into the database takes about 3 min on a 486 computer running under Windows™ '95, with 16 Mb RAM and an 1.2 Gb hard-disk. Printed reports can be generated as the decrypted file is processed. The time between starting downloading new results and accessing these results via the search screen is therefore minimal, and the Internet solution has proved to be very acceptable to the doctors, who are getting most samples turned around (i.e. analysed and reported) within the same day. Few health-care clinics would process more than several hundred patients a day, and so transmitting results in this way is an ideal solution for the clinic–laboratory interaction.

LIS-Interlink has proved to be a powerful marketing tool, as it can be easily demonstrated from any site in Poland (or the world) using a portable computer at the cost of a local phone call. Potential clients can easily see how they will interact with the Nova Medical Polska laboratory.

### Conclusions

The LIS-Interlink project was designed, developed and implemented in four months, and has been successfully installed and operating live for six months. LIS-Interlink is additionally being used as a marketing tool to expand Nova Medical Polska's laboratory business. The provision of local SQL databases for the clinics enables doctors to access cumulative patient data from any terminal on the local network (given appropriate security clearances), improving patient care. In this way they can easily track the patient's history and see if any untoward changes have occurred. Patient confidentiality is assured by use of appropriate high-security data encryption techniques, and Web server security. In future there is also a possibility of adding modules to LIS-Interlink in order to derive demographic statistics (for example reference intervals and distribution of cholesterol concentration in the local population). A remote Internet requesting module is currently under development to speed the BioLink™ registration of the many samples (100–200 currently) which arrive from the remote health centres each day. The local database also has fall-back security in case of hardware or software failure: all the results are stored in the main BioLink™ laboratory system and the laboratory can re-send any subset of the centre's data in order to rebuild the databases.

